# Sudden rupture of small aneurysm of the radial artery in a patient with COVID‐19 pneumonia

**DOI:** 10.1002/ccr3.4285

**Published:** 2021-06-23

**Authors:** Daniela Mazzaccaro, Matteo Giannetta, Giovanni Malacrida, Dino Zilio, Alfredo Modafferi, Paolo Righini, Massimiliano M. Marrocco‐Trischitta, Luca Vaienti, Giovanni Nano

**Affiliations:** ^1^ Operative Unit of Vascular Surgery IRCCS Policlinico San Donato Milan Italy; ^2^ Operative Unit of Plastic Surgery IRCCS Policlinico San Donato Milan Italy; ^3^ Department of Biomedical Sciences for Health University of Milan Milan Italy

**Keywords:** aneurysm, arterial rupture, COVID‐19, radial artery, radial catheter

## Abstract

In patients with COVID‐19, even small radial aneurysm may suddenly rupture.

## Graphical abstract

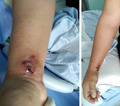

## 1 INTRODUCTION

We report the case of sudden rupture of a radial artery small aneurysm occurring in a 63‐year‐old patient who had been hospitalized for COVID‐19 pneumonia.

There is increasing evidence of vascular complications related to COVID‐19, including arterial and venous thrombotic events.^1^

However, reports about the occurrence of arterial rupture in patients hospitalized for COVID‐19 pneumonia are lacking.

These patients frequently require percutaneous arterial procedures, such as radial/brachial catheterization for invasive blood pressure monitoring or blood sampling for gas analysis. Therefore, they are at increased risk of developing vascular complications of the access site.

We report the case of a 63‐year‐old patient hospitalized for COVID‐19 pneumonia requiring noninvasive mechanical ventilation, who developed arterial rupture of a small aneurysm following arterial catheterization.

## 2 CASE DESCRIPTION

Patient's consent was obtained to the anonymous use of his clinical data for research purposes.

A 63‐year‐old man with a medical history of myocardial surgical revascularization was admitted to our hospital for a COVID‐19 pneumonia, which required ventilatory support until the need to wear a continuous positive airways pressure (c‐PAP) mask up to 60% FiO2 at day 4. Therapy with hydroxychloroquine 400 mg twice a day, lopinavir/ritonavir 400/100 mg twice a day and antibiotic coverage with ceftriaxone 2 g daily and azithromycin 500 mg daily was set up, according to the internal protocol. Moreover, a 4 French catheter (BD Medical^™^) was placed in his right radial artery for the arterial blood gas analyses, without any troubles. Then, he received a single dose of tocilizumab 640 mg and started methylprednisolone (40 mg twice a day). The respiratory function progressively improved until a complete weaning from the c‐PAP in day 10. The radial artery catheter was removed after 8 days in day 12 without any complications, and local compression was applied to the wrist. However, on day 14 a painful tumefaction in his right wrist, which extended to the forearm was found. The upper limb was warm, motility and sensitivity of the hand were preserved, and both radial and ulnar pulses were palpable. Doppler ultrasonography showed the presence of edema of the forearm extending to the arm, and the regular patency of the arterial and venous axis of the right upper limb, with regular flows and without any hematoma surrounding the artery, which had a regular caliber. A slight compression of the upper limb was then applied with a bandage.

**FIGURE 1:**
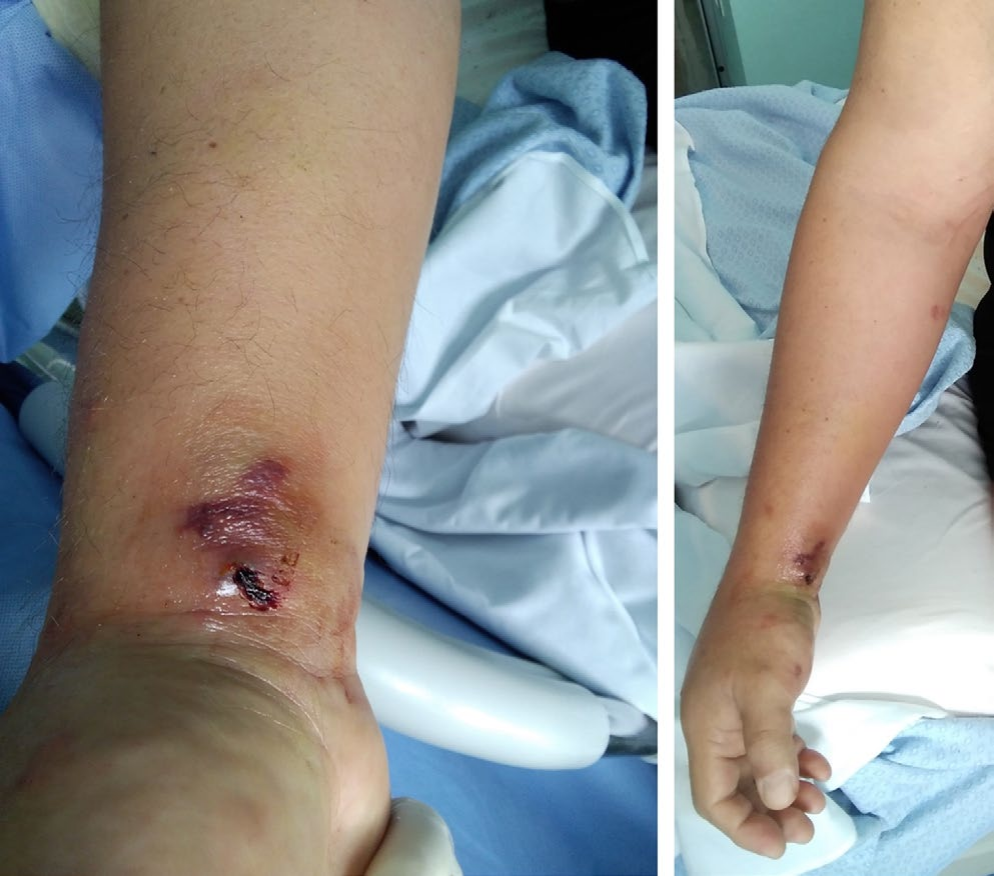
Pictures of the clinical presentation of the patient's right wrist (image on the left side). Note the edema extending to the forearm (image on the right side)

**FIGURE 2:**
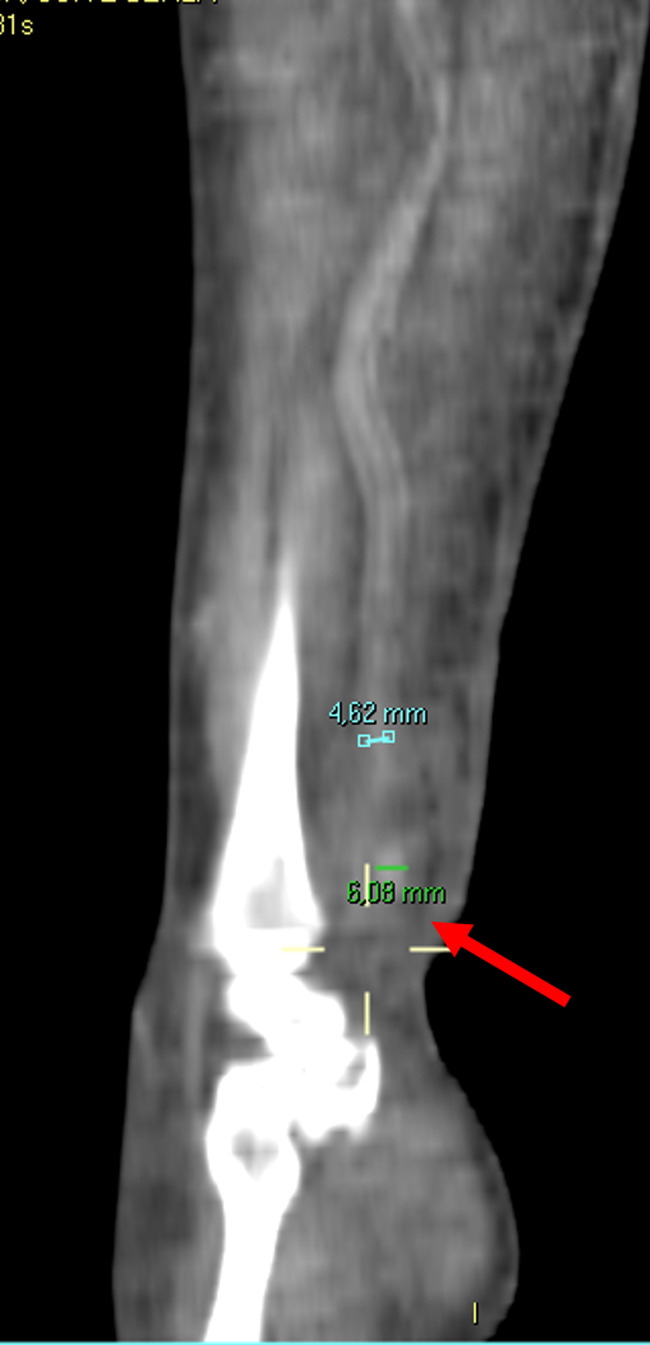
Right upper limb CTA showing a focal ectasia of the radial artery of 6 mm (red arrow), with edematous infiltration of the surrounding tissue and inflammation of the flexor muscles of the forearm

**FIGURE 3:**
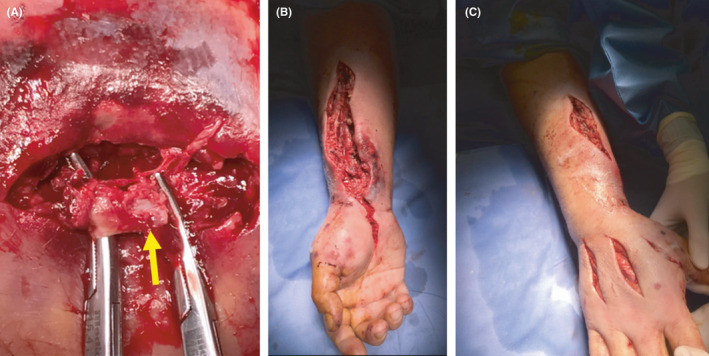
Intraoperative finding of laceration of the radial artery A. Note in figure B the fasciotomies of the flexor muscles' lodge at the level of the third middle‐distal of the forearm, and at the transverse ligament of the carpus. Figure C shows intermetacarpal fasciotomies on the back of the hand at I, II, and III space, with immediate decongestion of the edematous component

However, on day 16 the clinical picture worsened (Figure 1) and the patient complained of continuous pain of his forearm with initial impairment of the sensitivity and the motility of his right hand. Doppler ultrasonography was repeated, showing the presence of a small aneurysm of the radial artery at the wrist. The patient then underwent a right upper limb computed tomography angiography (CTA), which confirmed the presence of a focal ectasia of the radial artery of 6 mm (reference diameter of the artery above the ectasia was 4.5 mm), with edematous infiltration of the surrounding tissue and inflammation of the flexor muscles of the forearm (Figure 2). Blood cultures were negative; nevertheless, antibiotic therapy with daptomycin 10 mg/kg/day was set according to the infectivologist's suggestion, but 2 days later active bleeding from the right wrist suddenly occurred. Manual compression was unsuccessful; therefore, emergent surgery was performed. In the operating room, while keeping manual compression in the radial position at the distal third of the forearm, a right longitudinal incision was performed laterally at the wrist. A wide hematoma with active bleeding from the radial artery was found. The artery was completely lacerated (Figure 3A). Due to the impossibility of proceeding with arterial reconstruction, surgical ligation was performed with 5/0 polypropylene suture. Fasciotomies of the flexor muscles' lodge were also performed, in order to decompress the underlying median nerve which appeared to be edematous (Figure 3B). Adjunctive intermetacarpal fasciotomies on the back of the hand were performed at I, II, and III space, with immediate decongestion of the edematous component and good refill on all the fingers (Figure 3C). Intraoperative culture of both the endothelium and the surrounding tissue was negative for the presence of any bacterial agent.

The postoperative course was uneventful, with complete recover of sensitivity and motility of the hand. A duplex ultrasound was performed in the first postoperative day, showing adequate vascularization of the interdigital arteries, supplied by a regularly patent ulnar artery.

The patient was then discharged in good clinical conditions on day 28.

## 3 DISCUSSION

Radial artery catheterization is a common maneuver, which is usually performed for invasive arterial pressure monitoring,^2^ for endovascular and cardiac percutaneous procedures^3^ or when there is the need for frequent arterial blood gas determination, such as in COVID‐19 patients.

Indwelling radial artery catheters are generally safe, with an overall rate of associated vascular complications lower than 0.5%, being radial thrombosis the most reported.^4^

Furthermore, aneurysmal degeneration after radial artery catheterization has been reported in less than 0.1% of the cases,^5^ and radial artery rupture after arterial catheterization has been occasionally described.^6^

In our case report, the patient had a small radial aneurysm, which was, however, complicated by sudden rupture, despite antibiotic treatment and the not excessive size of the lesion. Some pathogenic mechanisms hypothetically could have contributed together to the development of such a complication.

First, the reduced local immune defenses following the administration of immunosuppressive drugs, such as hydroxychloroquine, tocilizumab, and corticosteroids, needed for the COVID‐19 pneumonia, may have contributed to a possible local infection, even if the intraoperative cultures of the endothelium were negative.

As a second issue, the SARS‐CoV‐2 infection itself may have played a role in the impairment of the arterial wall. Recently published papers have described a significant increase in vascular disorders in COVID‐19 patients.^1^ Furthermore, recent studies have shown direct viral infection of the endothelial cells through the angiotensin‐converting enzyme 2 receptor, and diffuse endothelial inflammation that can result in widespread endothelial dysfunction.^7^

Moreover, the so‐called “cytokine storm” that is typical of severe COVID‐19 infection also can cause arterial wall weakening and fragility which further cause arterial rupture or aneurysm formation.^8^

Therefore, vascular surgeons should be alert in patients with COVID‐19, bearing in mind that arterial complications may occur not only in the form of ischemic diseases but also as hemorrhagic complications following even small aneurysm.

## 4 CONCLUSION

Small radial aneurysms following arterial catheterization may be complicated by sudden rupture in patients with COVID‐19. Vascular surgeons should, therefore, be aware of such complication to prevent potentially serious consequences.

## CONFLICT OF INTEREST

None declared.

## AUTHOR CONTRIBUTIONS

DM: involved in study design, data collection, writing, critical revision, and final approval. MG: involved in data collection, writing, critical revision, and final approval. GM: involved in data collection, critical revision, and final approval. AM, LV, PR, MMT, and GN: involved in critical revision and final approval. All authors read and approved the final version of the manuscript.

## CONSENT FOR PUBLICATION

Patient's consent was obtained to the anonymous use of his clinical data for research purposes.
